# Maximising Societal Benefit From the Control of Neglected Zoonoses: Identifying Synergies and Trade-Offs in the Control of *Taenia solium*

**DOI:** 10.3389/fvets.2021.794257

**Published:** 2022-02-09

**Authors:** Cristina Soare, Amelia Garcia-Ara, Alessandro Seguino, Matthys Uys, Lian F. Thomas

**Affiliations:** ^1^The Royal (Dick) School of Veterinary Studies, University of Edinburgh, Midlothian, United Kingdom; ^2^School of Veterinary Medicine and Science, University of Nottingham, Nottingham, United Kingdom; ^3^Institute of Infection, Veterinary and Ecological Sciences, University of Liverpool, Neston, United Kingdom; ^4^International Livestock Research Institute, Nairobi, Kenya

**Keywords:** *Taenia solium*, one health, control, economic analysis, societal benefit

## Abstract

Interventions to control or eradicate neglected zoonoses are generally paid for through the public purse and when these interventions focus on the animal hosts, they are often expected to be performed and financed through the state veterinary service. The benefits of control, however, accrue across the human, animal, and environmental spaces and enhance both public and private interests. Additionally, disease control interventions do not take place in a vacuum and the indirect impacts of our actions should also be considered if the societal benefit of interventions is to be maximised. With the caveat that unintended consequences can and will occur, pre-identifying potential synergies and trade-offs in our disease control initiatives allows for them to be considered in intervention design and monitored during programme roll-out. In this paper, using a One Health approach with the example of *Taenia solium* control, we identify potential indirect impacts which may arise and how these may influence both our choice of intervention and opportunities to optimise the animal, environmental, and societal benefits of control through maximising synergies and minimising trade-offs.

## Introduction

Low and middle income countries (LMICs) carry the vast majority (98%) of the health and economic burden of endemic zoonoses (1) as well as the disproportionate burden from foodborne diseases (2). Making rational decisions around the allocation of scarce resources to control these diseases is assisted by economic analysis, an approach which seeks to “add value through a search for optimality” (3). In order to undertake such analysis a problem must first be identified and described, and the potential interventions compared for their cost-effectiveness (where a non-monetary “natural” unit of health is used as the outcome) or for their benefit: cost ratio (4–6). The control of zoonotic diseases is often paid for from the public purse, reflecting the public goods occurring from these interventions, and therefore when considering the control of zoonotic pathogens, a societal perspective to economic analysis may be considered most appropriate (5). If we wish to evaluate interventions according to their overall societal impact it is necessary to first identify the synergies and trade-offs which may occur in areas outside of the primary intervention target. Identification of these positive and negative “externalities” when designing interventions will allow for them to be monitored, potentially quantified and in the case of trade-offs, mitigate them when possible.

This paper outlines such an identification process using the example of the zoonotic parasite *Taenia solium*, the etiological agent of neurocysticercosis, one of the leading causes of acquired epilepsy in humans in endemic regions (7). This parasite is highly associated with marginalised communities where free-ranging pig production, poor sanitation coverage and lack of sufficient meat inspection converge allowing the lifecycle to propagate. The health burden, as measured by Disability Adjusted Life Years (DALYs) attributable to *T. solium* is considerable, and in the Africa-E sub-region, the sub-region in Africa with this highest childhood mortality burden (8), is estimated to be >176 DALYs/100,000 people (95% CI 134–229), making it the foodborne zoonosis with the highest health burden in this region (9).

**Figure 1:**
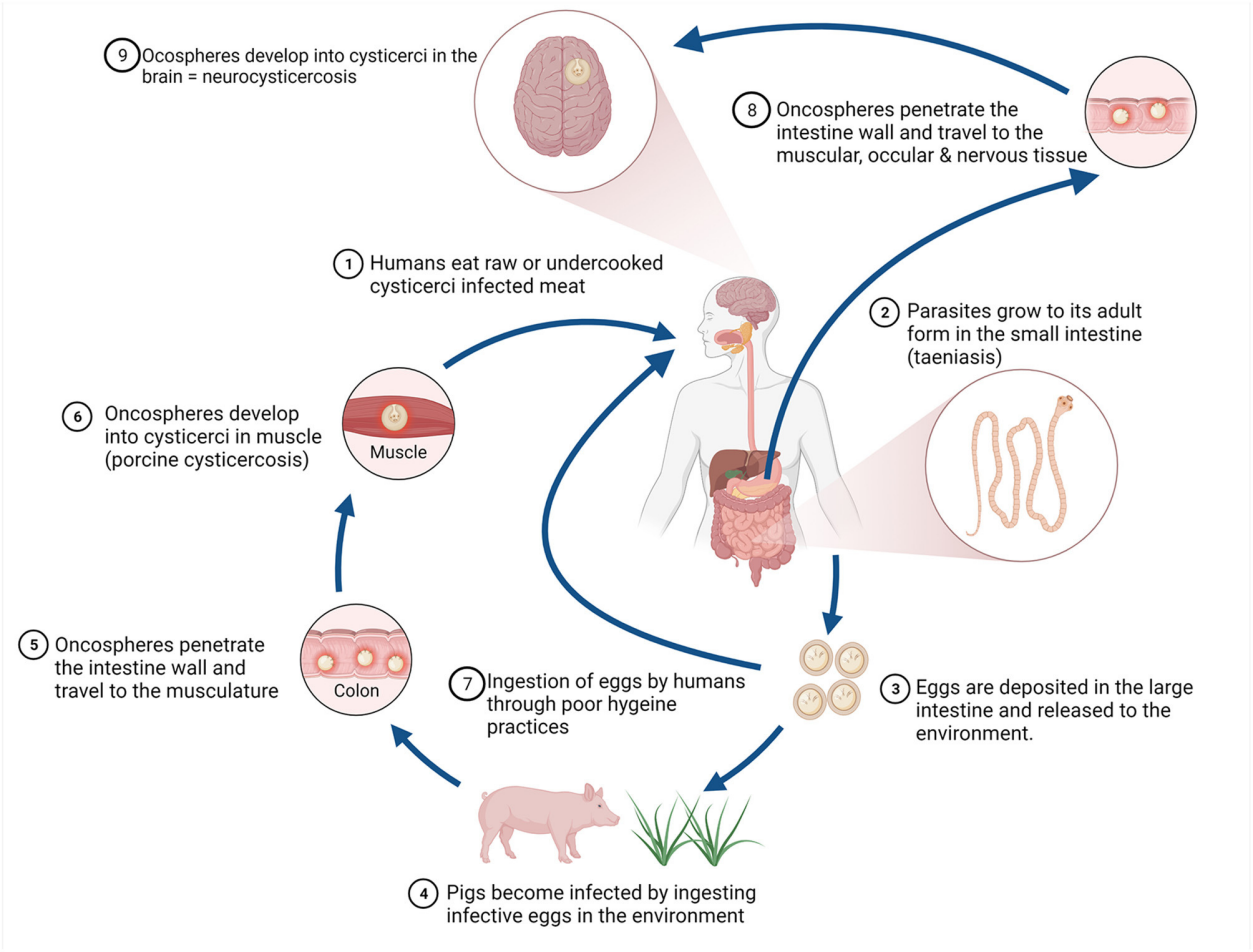
Life cycle of *Taenia solium*. Figure created with BioRender.com.

Domestic pigs are the main intermediate host of *T. solium*, with cystercerci in the musculature (porcine cysticercosis) and consumption of raw or undercooked pork containing the cysticerci leading to the development of the adult tapeworm in the small intestine of humans (taeniosis) (10). Humans shed tapeworm eggs in faeces, contaminating the environment where these may survive for up to 9 months (11). Taeniosis in humans is typically asymptomatic, with rare sequelae including bowel obstruction and gall bladder perforation (12). Substantial health burden is caused, however through the aberrant infection of humans with the intermediate stage of the parasite after consumption of the viable eggs (human cysticercosis). In humans, the cysticerci can form in the musculature, ocular tissue and in the central nervous system causing neurocysticercosis, inducing clinical signs such as epilepsy, headaches, signs of increased intracranial pressure and focal deficits (13). The lifecycle of *T. solium* is illustrated in Figure 1.

There is international advocacy for intensified control strategies for the management of *T. solium*, which is a target pathogen in the 2030 World Health Organization “Road Map” for control of Neglected Tropical Diseases (14). The success of Mass Drug Administration (MDA) for major neglected tropical diseases has largely been made possible through drug donations by pharmaceutical companies (15) and the recent announcement by Bayer that Praziquantel will be made available for national *T. solium* control programmes is an exciting step (16). To date, however, control programmes for this parasite, as reviewed systematically by Coster et al. (17) have almost entirely been driven by academic research. The scale-up and sustainability of programmes going forward requires appropriate finance mechanisms, with an appropriate cost-sharing structure between the human and veterinary health sectors and between the public and private sectors, as has been recommended previously for brucellosis and rabies control (18).

To provide a rationale for investment in such control programmes based upon objective prioritisation of budgetary allocations, pragmatic and robust impact evaluations of interventions are required. To identify benefits (synergies) or potential harms (trade-offs) related to *T. solium* control, we initially consulted two systematic reviews on the subject to create a list of potential strategies (17, 19) sometimes referred to as our “toolkit of options” (20). “Health Education” was not considered as a standalone intervention within this exercise, as we consider it to be an integral aspect of all described interventions, related as it is with the promotion of specific actions within the “toolkit.” With these options, which target different points in the parasitic lifecycle, in mind, we brainstormed to identify impacts of these options external to those on *T. solium* prevalence or incidence. The non-systematic approach taken to this identification process means that our framework may not be comprehensive but provides an example of the thought exercise which could be incorporated into intervention design for many pathogens.

## Identifying Synergies and Trade-Offs for Different Control Options

### Pharmaceutical Approaches in the Porcine Host

Highly effective pharmaceutical methods of preventing or treating *T. solium* in the porcine host have been developed. Oxfendazole (OFZ) at a single oral dose of 30 mg/kg has been recognised as being highly effective to treat the infection (21, 22) and is the drug of choice due to lack of negative effects, minimal cost and relatively short withdrawal periods (8–14 days). A porcine formulation of OFZ (Paranthic^®^ 10%) is now manufactured but only licenced for use in some African countries (23). Use of OFZ will not prevent reintroduction of the parasite, however. The vaccine TSOL18 has proved highly effective at preventing porcine infections, or preventing re-infection after OFZ treatment in several field trials (24–26). This vaccine is now under commercial production as Cysvax^®^ and has been licensed in several countries (27).

There is currently no evidence that porcine cysticercosis (PCC) in itself causes any visible reduction in productivity, and in countries where few disincentives exist for presenting infected pigs to slaughter, the willingness of farmers to pay for a vaccine appears to be low (28). With the balance of benefits from vaccination heavily tipped toward the public health sector, there would be a strong argument for public health provision or subsidisation of rolling out the vaccine. Opportunities exist however, to “bundle” the TSOL18 vaccine with others for production limiting diseases similar to a trial undertaken in Laos where the TSOL18 vaccine was rolled out alongside vaccination for classical swine fever (CSF) (29). Partial budget analysis of this intervention indicated a positive benefit: cost ratio to farmers, driven by the mitigation of production losses due to CSF (29).

In contrast OFZ has intrinsic private benefit to pig farmers through the synergistic impact on other endoparasites which have a negative effect on productivity, in particular the main nematodes occurring in pigs (*Ascaris suum, Strongyles* spp, *Oesophagostomum* spp, and *Trichuris suis*) (22, 30). Many studies of gastro-intestinal parasites of pigs raised under low-input systems in the countries in which *T. solium* is endemic have demonstrated a high prevalence of these infections (31–35), which will impact on feed conversion efficiency and kill-out percentage, translating into a real constraint on their pork production enterprises (36). Demonstrating to farmers the financial benefits of adopting OFZ treatment in their pigs has the potential to improve the willingness to pay (WTP) for this control option.

The use of anthelmintic treatments does however come with potential negative consequences, in this case the potential human health impacts due to the presence of residues in meat, development of anthelmintic resistance and ecotoxicity from residues accumulating in the environment.

Concerns over potential toxicity or hypersensitivity in humans consuming meat containing drug residues led to the setting of maximum residue limits (MRL) for drugs licensed for veterinary use. The pharmacokinetics of different drugs informs the time which must elapse (withdrawal time) before meat from treated animals is fit for human consumption. The benzimidazole family of which OFZ is a member appear to be stable in meat even after cooking (37), so residues present at slaughter are highly likely to be ingested at consumption. Poor enforcement of residue limits within the resource-constrained settings in which *T. solium* is endemic, leave open the potential that meat may be consumed with residues over the MRL (38).

As the benzimidazole class is widely used in veterinary and human medicine, the development of anthelminthic resistance to OFZ must also be considered as a potential negative consequence of intensified use for *T. solium* control (39). The extensive systems in which many pigs are raised in endemic areas may slow the selection pressure in the parasite, but consideration should be made of this potential when intervention programmes are developed to avoid the resistance issues already faced by the ruminant livestock sector (40, 41).

Ecotoxicity from compounds of the benzimidazoles has been demonstrated in aquatic and terrestrial organisms (42, 43). Drugs from this class have been demonstrated to be excreted in faeces and urine predominately in an unaltered, active state, and that these compounds can persist in porcine faecal material for periods of a hundred days or more after excretion (44). Consideration of the ecosystem services of organisms impacted by anthelmintic residues is an important aspect of any impact evaluation from a truly societal perspective, and risk mitigation measures should be considered to protect the environment, with particular care being taken to avoid water contamination from the dung of treated animals (45).

### Porcine Husbandry Interventions

The OIE Terrestrial Animal Health Code for the control of PCC does not consider pharmaceutical treatments, and instead focuses on farm husbandry approaches which prevent the direct and indirect exposure of pigs to untreated human faeces (46). There is an evident correlation between husbandry practices and the risk of PCC, with studies demonstrating a significantly higher sero-prevalence in extensively kept pigs compared to those raised in more intensive conditions due to increased transmission opportunities from environments contaminated by eggs (47–49).

Confinement of pigs to restrict their access to contaminated soil and water reduces the risk of acquiring PCC as well as other pathogens of public health and production importance. The improved biosecurity resulting from pig confinement reduces the potential for transmission of African swine fever (ASF), a virulent viral infection with high levels of mortality amongst infected pigs which can be spread by direct contact with an infected pig or warthog, through the bite of an infected tick or via contact with fomites carrying the virus (50, 51). ASF is an important production-limiting disease across sub-Saharan Africa where *T. solium* is endemic and risk reduction for ASF may be an incentive to farmers to adopt improved practices. Confinement of animals and the provision of supplementary feed also provides an opportunity to improve average daily weight gain, thereby shortening the time taken to raise a pig to slaughter weight which may result in improved gross margin for the pig production enterprise.

The profitability of small-holder pig farming enterprises, however, is often based upon narrow margins and demonstrates a significant influence from the cost of feeds, with a study in Kenya indicating that a 1% increase in feed costs had the potential to reduce pig enterprise profitability by 25% (52). This demonstrates a potential risk to farmer livelihoods when previously low-input enterprises are moved into a confined system which becomes highly reliant on this one key input. Further research is needed on farm enterprise economics to provide data to farmers on the potential monetary return on investment from enhanced husbandry practices including confinement, appropriate feeding, and biosecurity.

Making changes in a livestock enterprise which require additional labour inputs, for the collection of feeds, cleaning of pens etc, can also alter the inter-household gender distribution of labour. There are examples of these additional tasks falling predominately onto the women in the family, particularly where small-stock are concerned (53), potentially reducing the time available for other opportunities inside or outside the household (54). If the finances generated by a livestock enterprise remain in the control of a female household head there is evidence that this may increase the nutritional outcomes of the children in that household (55). Despite a reliance on female labour to care for confined pigs, as a livestock enterprise commercialises, the control of the enterprise may pass to the male head of the household. In these cases, the women not only lose control of the money generated by the enterprise but may not have sufficient agency in the household to request for veterinary inputs, or make other important management decisions (56), reducing effective livestock management based on daily observations of the animals. Understanding the intra-household gender dynamics and ensuring that changes in the livestock enterprise are made in a way which acknowledges and preferably seeks to transform these dynamics is paramount.

Trade-offs in shifting small-holder pig production from a free-ranging to a confined production model also include potential detriments to animal health and welfare and environmental concerns. Carriage and shedding of key microbial pathogens, such as *Salmonella* and *Campylobacter* spp., can be exacerbated under confined conditions, in turn increasing the risk to consumers of acquiring these foodborne diseases and requiring close monitoring for the protection of public health (57, 58).

Careful attention must also be given to the appropriateness of pig housing to avoid animal welfare and health problems. Although consideration of animal welfare is a relatively new area of concern within many endemic countries, there is evidence of consumers' increasing interest in the topic and a willingness-to-pay for improved welfare in livestock production systems has been documented in Kenya, indicating an economic driver for ensuring high welfare standards (59, 60). There is also an argument that economic evaluations from a societal perspective should explicitly consider and value animal welfare as a social welfare function (61). The OIE terrestrial code establishes animal welfare specific recommendations for pig keeping which can be adapted to the endemic settings (62).

The location of confined pig production in relation to its impact on land-use changes, and proximity to habitat for high-potential zoonotic disease hosts such as bats and rats is an important consideration, as demonstrated by the concurrent intensification of mango and pig farming in the Malaysian peninsula in the late 1990's which resulted in the spillover of Nipah virus into humans (63). Human-to-human transmission of the virus, which causes neurological symptoms and has a high fatality rate, has now been identified in Bangladesh, subsequent to multiple independent spillover events driven by land-use change (64).

Environmental externalities may also arise from confinement of livestock, including the land and water footprint of growing additional crops for pig feed and the potential leachate from pesticide, herbicide and fertiliser used on these crops. The environmental impact of pig manure will depend upon the production system adopted and the manure management strategies applied. Water contamination with faecal material introduces pathogens and antimicrobial resistant bacteria/ pathogens into the environment which may be transmitted to other animals or man. Drug residues have the potential to be toxic to the aquatic ecosystem whilst nitrates in manure lead to eutrophication and the death of aquatic organisms and those which rely on them (65). Pig manure releases the greenhouse gases (GHG) nitrous oxide (N_2_O), methane (CH_4_) and carbon dioxide (CO_2_) and also causes a public nuisance from odour (66). The increased density of pigs kept under a confined system, particularly when combined with industrial agglomeration can lead to high environmental impacts in these areas. The spatial aggregation of pork production in high income countries has been demonstrated to improve the profitability of individual farms, and it could be expected that as the pig industries in LMICs intensify, similar agglomerations will occur as farms cluster around the source of feed provision or in localities with strong market demand.

Appropriate waste management strategies will be required to mitigate these impacts if farmers are to be encouraged to move toward intensified systems which in turn is likely to necessitate a strong regulatory framework. Best practice manure management may not only mitigate the negative environmental externalities of changes in husbandry practices, but may also result in private sector benefits where appropriately treated manure is spread in appropriate quantities on crop land, or utilised for renewable electricity production if capital is available (67).

### Interventions Relating to Food Safety Legislation

The key legislative requirements relevant to the control of *T. solium* are the regulations relating to the inspection of meat products. Ante- and post-mortem inspections conducted at abattoirs aim to protect both animal and human health by preventing, detecting and controlling hazards originating from animals (68). This process provides one of the key synergies between control of zoonoses and improvements in food safety. Along with *Taenia solium* (69) several zoonotic diseases present within sub-Saharan Africa may have either clinical signs or detectable lesions at inspection including tuberculosis (*Mycobacterium bovis*) and *Ascaris suum* infection (70–72).

Meat inspection also serves as an important source of surveillance and a detection point for contagious and production animal diseases, allowing appropriate, timely control activities to be conducted. These diseases include African swine fever, classical swine fever, and foot-and-mouth disease (71). The early detection and control of contagious disease is especially important for small-holders, in order to protect farmer livelihoods and financial security within vulnerable communities (73). The meat from pigs slaughtered at a registered abattoir complying with the relevant legislation and meat inspection, are usually subject to more hygienic slaughter practices and are at a lower risk of foodborne bacterial contamination (74). Additionally, the diagnosis of pathological and welfare conditions by trained personnel during abattoir inspection can serve as an important source of information to the farmer in order to improve animal health, production and welfare (75). Aiming for health maximisation through the rectification of disease conditions can lead to an increase in herd well-being and productivity and to a decrease in losses incurred by the farmer (76).

“Traditional” meat inspection, reliant on visualisation, palpation and incisions and as practised in the majority of *T. solium* endemic countries is, however, relatively insensitive in detecting cysticerci (77) and has no efficacy in relation to microbial hazards (78). The process of palpation and incisions can be time-consuming for the inspector whilst acting as a source of cross-contamination of the carcass by microbial pathogens (78, 79). Freezing of infected carcasses at −20°C for 1–3 days has been demonstrated to be successful in killing cysticerci (80). However, in many of the regions where the parasite is endemic, the infrastructure for this may not be readily available, while the process can also reduce the value of the carcass, and may render the meat unacceptable to consumers who prefer fresh meat (81). The enforcement of meat inspection regulations and subsequent condemnation or downgrading of meat can drive infected meat into the informal “black” market, exacerbated by the poor enforcement of legislation, inadequate numbers of veterinary public health officials, and periods where the demand for meat is high (82). Pigs may be lingually examined for *T. solium* cysts by traders prior to purchase and slaughter, and positive animals illegally slaughtered or sold at a lower price (83, 84). These informal markets have the potential to reduce the financial risk to farmers and traders, as they provide a conduit for selling meat which would otherwise be condemned, but they directly reinforce inequity in access to food safety where the poorest consumers continue to be exposed to food safety hazards which richer consumers are protected from (74). The education of consumers is essential, as these practices are unlikely to be contained if the high demand for illegally slaughtered meat persists (82).

### Pharmaceutical Interventions in the Human Host

The use of mass drug administration (MDA) in human populations at risk of infection is a mainstay of control programmes for neglected tropical diseases, including soil transmitted helminths (STH), schistosomiasis, lymphatic filariasis, onchocerciasis and trachoma, and over a billion people a year are currently treated across Asia, Africa and Latin America. These programmes have demonstrated dramatic reduction in disease burden, both for their intended targets and for many additional diseases which were unexpected targets at programme inception (85). The integration of vertical, single disease focused interventions into interventions for multiple diseases, or within wider health system services will provide opportunities for improved economies of scale and scope (86).

Praziquantel at 40 mg/kg is effective against both *T. solium* and schistosomiasis (87), whilst a triple dose of 400 mg albendazole is effective against *T. solium* and STH (88). Understanding co-endemicity of these parasites is therefore important to guide the best choice of pharmaceutical agent in order to enhance the synergies of MDA programmes. These synergies can be captured quantitatively through consideration of the DALYs averted through MDA. In Laos PDR the cost-effectiveness of the MDA component of a combined human-pig intervention was strongly driven by the treatment of STH which was causing widespread morbidity in the community (29). Many additional benefits have been indicated to accrue from the mass treatment of gastro-intestinal parasites including; improved weight gain, improved school assessment scores and even improved labour market outcomes later in life as reviewed in 2017 by Ahuja et al. (89).

Potential negative externalities of widespread anthelmintic use in human populations include social mistrust, eco-toxicity, anthelmintic resistance, and potential adverse reactions. Praziquantel crosses the blood-brain barrier, and the potential for its use to trigger epilepsy in latent neurocysticercosis sufferers is being closely monitored by those conducting MDA programmes (90). Anthelmintic resistance has not yet been reported in the large MDA programmes already running for schistosomiasis and STH, but monitoring should nonetheless continue (91). Ecotoxicity has been discussed under porcine pharmaceutical interventions but is an under-studied area within the context of MDA for NTDs. The ethics of MDA have been questioned on occasion and the potential to cause social unrest and mistrust of the health care system has been documented (92) and some of the stated benefits of school-based programmes are under debate, with more evidence required to monitor and quantify them (93).

### Water, Sanitation, and Hygiene (WASH) Related Interventions

Other potential interventions for *T. solium* targeted at the human host include the provision of improved sanitation infrastructure and of appropriate and context-specific health education messages related to sanitation, personal hygiene, and safe food preparation. In vulnerable communities of sub-Saharan Africa, although sanitation has improved over the last two decades, hand washing facilities are absent or deficient in 75% of households, 39% don't have access to safely managed drinking water and open defecation is still practised in ~70% of the population (94). Open defecation results in propagation of the tapeworm cycle whilst inadequate hand washing facilities and unsafe drinking water are contributing factors to human cysticercosis (95).

Improving societal sanitation and hygiene through increased latrine and potable water coverage and education on safe food preparation potentially has the opportunity for the greatest added value amongst any of the interventions discussed, due to the protective effect on many other pathogens, including diarrheal agents. Diarrheal diseases are responsible for one of the highest burdens of disease across LMICs, accounting for 1 in 9 child deaths worldwide, with more children dying on a daily basis from diarrheal pathogens than from AIDs, malaria and measles combined (96). Whilst rotavirus vaccination and improvements in breastfeeding rates have been responsible for some of the decrease in burden from diarrheal diseases in the last 20 years (97), there is a consensus that WASH programmes including the adoption of systems for treating and storing drinking water, health education and latrine provision have made cost-effective contributions to this decline (98). The United Nations have recognised that clean water and sanitation are a basic human right, and the public health protection endowed by WASH services, enables a productive and prosperous society, indicating that the strong correlation between Human Development Index and WASH service provision may be self-reinforcing rather than a uni-directional relationship (99).

Despite the potential for different WASH interventions to disrupt *T. solium* transmission, only two control trials to date have attempted to monitor the impact specifically on this parasite (100, 101). In Burkina Faso the intervention appeared effective in reducing active human cysticercosis prevalence in one of the two study districts, demonstrating the potential for WASH interventions to be part of intensified control of *T. solium*. In Zambia, the programme failed to achieve sufficient latrine usage within the target community for a variety of reasons including cultural taboos related to who can have latrine access, and the intervention failed to make an impact on the prevalence of *T. solium* (101, 102). Yet the strong rationale for increasing basic sanitation levels as an integral aspect of sustainable development is undeniable.

Careful planning is required in order to minimise any potential negative externalities of such programme in terms of environmental contamination, odour, or public nuisance. Accounting for socio-cultural taboos regarding sharing of latrine facilities (102), and the need to ensure safety of facilities is also important to ensure equity in access across age and gender groups (103). If appropriate sewage treatment facilities are not available or suitable for the context, night-soil may be collected for use of fertiliser. Although this product offers large soil fertility benefits, the presence of potentially pathogenic microbes including viable *T. solium* eggs in this night-soil, requires that the product is carefully stored and treated prior to utilising it on pasture-land or plantations where pigs could acquire access (104).

## A One Health Framework to Identify, Monitor, and Quantify the Synergies and Trade-Offs of Zoonotic Disease Control

As a trans-disciplinary framework for solving complex problems across the human, animal, and environmental interface, we consider that the logical conclusion of a One Health approach is the evaluation of interventions from a societal perspective, aiming to maximise net societal benefit. As described here through the example of *T. solium* control, disease control interventions may provide both positive and negative externalities, “synergies and trade-offs” to a range of stakeholders. It is only through identifying these potential synergies as well as the negative impacts which may occur that the appropriate baseline and post-intervention monitoring can occur. Table 1 summarises the externalities we have described in this manuscript and indicates potential areas to monitor or mitigate. We have drawn these examples from our own brainstorming sessions and therefore cannot state that we have comprehensively identified all potential externalities.

**Table 1:** Summary of control strategies with potential synergies and trade-offs.

**Strategy**	**Potential synergies**	**Potential trade offs**	**Activities to enhance synergies & mitigate trade-offs**
Porcine anthelmintic ± vaccine	Reduced GI parasite burden, improve weight gain & farm profitability **Monitor:** Faecal egg counts, daily weight gain & farm enterprise profitability	Anthelmintic resistance, hypersensitivity reactions in humans, ecotoxicity to aquatic or terrestrial spp. **Monitor:** Monitor resistance levels, residues in meat, ecological monitoring of appropriate indicator species	Provide appropriate extension services to enhance husbandry & health care practices including rational anthelmintic use Bundle TSOL18 vaccine with context appropriate vaccines for production limiting diseases Disseminate farm enterprise profitability data to stimulate investment and identify “champion” farmers as advocates Enhance meat inspection to incentivize production of “clean pigs” and instigate residue testing
Confinement of pigs with appropriate supplementary feeding	Reduced disease transmission from roaming pigs, improved weight gains & farm enterprise profitability **Monitor**; Incidence of clinical episodes, daily weight gain & farm enterprise profitability	Animal welfare breaches from inappropriate housing, tight tethers, insufficient feed & water provision, disease transmission from overstocking/poor ventilation. **Monitor**; On farm or at slaughter welfare assessments including lung scoring at slaughter. On farm incidence of disease Environmental contamination from manure. **Monitor**; Manure management practices, GHG emission intensity and water contamination Increased reliance on women's labour without commensurate benefits to women. **Monitor**; inter-household labour and resource allocation	Provide appropriate extension services to enhance husbandry & health care practices including education on locally available feeds and ration formulation, pen construction and manure management practices Improve access to animal health provision Disseminate farm enterprise profitability data to stimulate investment and identify ‘champion' farmers as advocates Incorporate gender transformative approaches in intervention design
Meat inspection	Improved control of zoonoses, foodborne disease and transboundary animal diseases. **Monitor**; Reports and condemnations from meat inspectors	Economic shock to resource poor farmers or traders on condemnation of meat. **Monitor**; Number of condemnations, Stimulate an informal ‘black' market for sub-optimal meat **Monitor**; covert operations by law-enforcement to identify extent of black market Threat of retaliation for meat inspector. **Monitor**; perception of inspector of their ability to perform their jobs Bacterial cross-contamination from incisions. **Monitor**; Monitor microbial contamination of meat.	Provide farmers with the tools and agency to raise ‘clean' pigs Educate consumers to demand inspected meat (knowledge of health mark stamps etc) Investment to ensure full complement of staff, with regular training and provision of mobile phone reporting tools and facilitate use Empower meat inspectors to condemn unfit meat and provide law enforcement backing Monitor relative burden of parasitic vs microbial FBD and develop traceability options to enable risk-based approaches to inspection
Human anthelmintic treatment	Reduced burden of schistosomiasis and soil transmitted helminths leading to improved health and educational outcomes. **Monitor**; Prevalence of other parasitic infections, school attendance and attainment	Latent NCC may be stimulated **Monitor**; closely for adverse drug reactions Anthelmintic resistance **Monitor**; resistance profiles of targeted parasites Terrestrial and aquatic ecotoxicity **Monitor**; population of key indicator species Community unrest and resistance to programmes. **Monitor**; refusals to participate in programmes	Plan treatment programmes using co-endemicity maps to ensure most appropriate treatment regime. Undertake screening for potential NCC and adjust PRZ dose appropriately and Use Mass Drug Administration programmes only where necessary. Enhance latrine provision to reduce environmental contamination. Ensure a careful, culturally appropriate sensitisation programme with regular community consultation
Water, sanitation, and hygiene interventions	Reduced burden of diarrheal diseases. **Monitor**; incidence and burden of diarrheal disease Utilisation of night soil for fertiliser or biogas generation **Monitor**; number of households with composting latrines or biogas generation	Fear of breaking taboos, violence or injury **Monitor**; latrine use as well as coverage Use of night-soil as fertiliser may spread pathogens **Monitor**; treatment time and temperature and viability of pathogens before use on crops	Initiate with appropriate anthropological engagement with community to ensure latrine construction adheres to local cultural context and that access to latrines is safe and equitable Provide a strong sensitisation programme on benefits of WASH programmes. Utilise Community led total sanitation to enhance community uptake Provide extension services to promote alternative night-soil uses and ensure night-soil is fully treated to kill pathogens before use as fertiliser

The most appropriate intervention for any one pathogen will be context specific. The contextual factors for consideration within *T. solium* control have been summarised by Ngwili et al. (105), and include the epidemiological, socio-economic, cultural, historic, geographical and climatic context as well as considering aspects of institutional capacity including the presence of appropriate legislature, resource and political will. When considering the epidemiological context, the identification of additional “secondary” disease targets highlights the need to appropriately understand the co-endemnicity of different pathogens. In the case of *T. solium* for example, a high degree of co-endemicity of schistosomiasis or STH may favour a MDA approach in the human host, while the presence of production-limiting diseases of pigs may favour the potential to bundle a contextually relevant porcine vaccine alongside Cysvac^®^, such as the combination with classical swine fever vaccine in Lao PDR (29). Where the burden of *T. solium* is high, enhanced meat inspection techniques with targeted palpation and incisions may be the most appropriate method to support ongoing control. When the balance of burden shifts so that microbial hazards such as *Salmonella* spp., *Campylobacter* spp. and *Yersinia entrocolitica* become dominant, a risk-based approach with reduced incisions and opportunities for cross-contamination, may become most appropriate, requiring the presence of robust traceability systems (106).

In the majority of *T. solium* endemic communities pig production systems are poorly developed with consequent low productivity (107), whilst open defecation is practised by communities due to low or inappropriate latrine provision (102, 108). Given the potential to mechanically disrupt parasite transmission and the potential for high-value synergies with other human health, food security and economic development programmes by addressing these issues, we strongly recommend a heightened focus on these areas whilst ensuring that mitigation measures for potential trade-offs are designed in at conception.

Designing appropriate extension packages to promote best practice in animal health, feeding, environmental management, whilst optimising the gender equity, and animal welfare is a complex task and promoting adoption even harder. A thorough understanding of people's motivations for engaging with pig production, their financial and societal constraints and aspirations is needed. Incentives for engagement may be financial, requiring evaluation and dissemination of farm enterprise budget data or may be through increased social capital, due to societal recognition of good, “clean” pig production. Identification of local “champion” farmers, those managing their pigs under sanitary conditions whilst enhancing animal welfare and environmental protection through use of best practices, would provide an opportunity to promote such practices to other pig farmers within a similar context.

Legislation may also play a role in motivating farmers to improve production, for example enhancement of meat inspection services and the risk of condemnation of pigs may stimulate the uptake of pharmaceutical interventions (109). Understanding the way in which the pork value chain operates, the degree of integration and the governance structures can allow for interventions to be embedded in a systems approach and allow evaluation across different actors. Ex-ante modelling of ASF control options which incorporated enhanced biosecurity alongside the development of an integrated business model where farmers were integrated into a co-operative with which dedicated traders interact. Whilst implementation of biosecurity by farmers reduced ASF outbreaks, the profitability of the pig enterprise was projected to be compromised by the intervention, whereas the combination of a market-based intervention alongside improved biosecurity improved the profitability of all actors in the value chain, whilst stabilising the supply and price of pork to the consumer, demonstrating the utility of such a systems-based approach (110).

Evaluating interventions which create impacts across multiple dimensions is challenging. Economic evaluation approaches which can be used both *ex-ante* and *ex-post*, such as cost-benefit analysis (CBA) or cost-effectiveness analysis (CEA) require standardisation of costs and benefits, either into monetary terms (CBA) or by quantifying outcomes in appropriate non-monetary units (CEA). Within health care CEA using a non-monetary health metric combining mortality and morbidity such as the Quality Adjusted Life Year (QALY) or Disability Adjusted Life Year (DALY) is a mainstream approach and is mandated in several countries to provide justification for public investment in health technologies. Two approaches have been suggested to combine human and animal health outcomes, either into monetary terms for CBA or into a combined metric for CEA—the zoonoses-DALY (111, 112). It would appear that neither approach precludes combining additional impacts such as changes in ecosystem services, though the complexity, and difficulty in providing quantitative estimates of impacts may preclude their use. An alternative approach for decision making, often used to aid complex investment decisions is multi-criteria decision analysis (MCDA). Various MCDA are available which can include qualitative as well as quantitative data and their use is increasing in the realm of health policy (113). MCDA has been used to assist stakeholders in evaluating options for Lyme disease control, using an semi-quantitative assessment of impact across five critical domains, being; Public Health, Animal & Environmental Health, Social Impact, Strategic & Operational criteria and surveillance criteria (113). The MCDA provided a transparent process for decision making in which the weighting of criteria by stakeholders provides an explicit expression of the values stakeholder's place upon different impact domains.

We acknowledge that whilst designing interventions we may never fully anticipate all unintended consequences and attempting to do so may result in paralysis. We do consider, however, that whilst there is a need to provide “boundaries” to our problems, identifying positive, and negative externalities of our actions provides us a framework within which a broader societal perspective can be taken in our design and evaluation of interventions. We recommend further consideration of expanded economic evaluation frameworks suitable for tackling problems at the animal, human, environmental interface, or the further adoption of multi-criteria decision analysis in the field of zoonoses control. In this paper we have described many broader impacts relating to *T. solium* control and we hope this stimulates consideration by those designing control trials to expand intervention monitoring across these different domains.

## Conclusion

In conclusion, appropriate monitoring of intervention impacts is difficult and time consuming, particularly when these impacts fall across different sectors. We recommend programmes start by identifying key potential synergies and trade-offs so that they can be supported to look outside the primary target of a campaign into areas where societal benefit can truly be maximised and, where possible, quantified. We also recommend the development of appropriate One Health economic evaluation frameworks, integrating animal and human health, environmental economics and multi-criteria analysis to aid decision making and guide appropriate resource allocation to zoonotic disease control interventions.

## Author Contributions

LT: conception. CS, AG-A, AS, MU, and LT: first draft. All authors read and approved the final draft of the manuscript.

## Funding

LT was supported by the University of Liverpool-Wellcome Trust Institutional Strategic Support Fund, the Soulsby Foundation (https://soulsbyfoundation.org/) and the German Federal Ministry for Economic Cooperation and Development through the One Health Research, Education and Outreach Centre in Africa (OHRECA). Open access publication fees are supported by the University of Liverpool institutional access fund.

## Conflict of Interest

The authors declare that the research was conducted in the absence of any commercial or financial relationships that could be construed as a potential conflict of interest.

## Publisher's Note

All claims expressed in this article are solely those of the authors and do not necessarily represent those of their affiliated organizations, or those of the publisher, the editors and the reviewers. Any product that may be evaluated in this article, or claim that may be made by its manufacturer, is not guaranteed or endorsed by the publisher.
